# Changes in Adipose Tissue Distribution and Association between Uric Acid and Bone Health during Menopause Transition

**DOI:** 10.3390/ijms20246321

**Published:** 2019-12-14

**Authors:** Gloria Bonaccorsi, Alessandro Trentini, Pantaleo Greco, Veronica Tisato, Donato Gemmati, Nicoletta Bianchi, Melchiore Giganti, Maurizio Rossini, Giuseppe Guglielmi, Carlo Cervellati

**Affiliations:** 1 Department of Morphology, Surgery and Experimental Medicine, University of Ferrara, 44124 Ferrara, Italy; gloria.bonaccorsi@unife.it (G.B.); pantaleo.greco@unife.it (P.G.); melchiore.giganti@unife.it (M.G.); 2 Menopause and Osteoporosis Centre, University of Ferrara, 44124 Ferrara, Italy; 3 Center of Gender Medicine, University of Ferrara, 44121 Ferrara, Italy; donato.gemmati@unife.it; 4 Department of Biomedical and Specialist Surgical Sciences, Section of Medical Biochemistry, Molecular Biology and Genetics, University of Ferrara, 44121 Ferrara, Italy; alessandro.trentini@unife.it (A.T.); nicoletta.bianchi@unife.it (N.B.); 5 Department of Morphology, Surgery and Experimental Medicine, and LTTA Centre, University of Ferrara, 44121 Ferrara, Italy; veronica.tisato@unife.it; 6 Department of Morphology, Laboratory of Nuclear Medicine, Surgery and Experimental Medicine, University of Ferrara, 44124 Ferrara, Italy; 7 Rheumatology Unit, Department of Medicine, University of Verona, 37134 Verona, Italy; maurizio.rossini@univr.it; 8 Department of Clinical and Experimental Medicine, Foggia University School of Medicine, 71121 Foggia, Italy; giuseppe.guglielmi@unifg.it

**Keywords:** uric acid, purine metabolism, body fat mass, menopause transition, android fat distribution

## Abstract

Despite convincing experimental evidence, epidemiological studies on the effects of serum uric acid (SUA) on bone health are still conflicting since factors influencing SUA bioavailability have not been adequately considered. To shed some light on this issue, we investigated the impact of adiposity and menopause status on the relationship between SUA and bone health. We examined SUA in relation to bone mineral density (BMD) at different skeletal sites and with markers of bone metabolism in 124 pre-menopausal and 234 post-menopausal women and assessed whether adiposity, evaluated by anthropometry and dual x-ray absorptiometry (DXA), might have a discriminant role. After conservative adjustment (covariates: age, hormones treatment, smoking and time since menopause), SUA showed a significant and positive association with total hip BMD (*β* = 0.220, *p* < 0.01) among postmenopausal women, maintained also after adjustment for legs adiposity. Notably, stratification for waist circumference quartiles revealed that the correlation between SUA and total hip BMD was significant (*r* = 0.444, *p* = 0.001) in the highest quartile (91–100 cm). Our results suggest that SUA might be beneficial for bone health in postmenopausal women being characterized by a more android fat distribution, ascribing to SUA a discriminant role during menopause transition, potentially relevant also for men.

## 1. Introduction

Osteoporosis represents one of the major public health issues and despite the important progress in the knowledge, the pathogenic mechanism underlying osteoporosis development is still unclear and it remains the subject of a number of studies [1]. A considerable part of research efforts has been focused on the potential pathogenic role of oxidative stress (OS) in osteoporosis, in particular in the disease form occurring in women after menopause (i.e., postmenopausal osteoporosis) [2,3,4,5].

Convergent experimental studies have shown that reactive oxygen species (ROS) defined as prominent pro-oxidant substances, are able to stimulate the resorption activity of osteoclasts, and reduce at the same time the life-span and bone formation activity of osteoblasts [6,7]. Conversely, antioxidants appear to contribute to the activation of osteoblast differentiation, the mineralization process and decrease at the same time osteoclast activity [8]. Preclinical findings are partially corroborated by clinical/epidemiological studies. In particular, data on the relationship between serum/plasma markers of OS, including antioxidants, are highly variable and no firm conclusions have been achieved so far [5,9,10,11].

A paradigmatic example in this context is serum uric acid (SUA), the most abundant non-enzymatic endogen antioxidant present in systemic circulation [12]. While recent studies have shown that SUA levels are positively correlated with bone mineral density (BMD) in the general population [13,14,15], contrasting results have been reported suggesting no associations between SUA and bone health markers [16], or marked differences in correlation strength and direction between men and women [17].

Overall, conflicting results in epidemiological studies such as those previously mentioned, are often due to unmeasured or inadequately measured sex-specific confounding factors, as well as a difference in study cohorts structure. In epidemiological studies focused on the relationship between SUA and bone health, indexes of body adiposity could be important confounders, being well-documented correlates of both purine catabolite and BMD. In particular, surrogate markers of visceral obesity, ranging from waist circumference (WC) to the more accurate indexes measured by dual x-rays absorptiometry (DXA) or computer tomography, are stronger predictors of SUA and BMD than body mass index (BMI) [10,18,19].

Besides adiposity amount and distribution, other possible interfering factors should be taken into account in these types of studies. In particular, it is well recognized that sex and hormones (in primarily estrogen) as well as age, influence SUA levels [16]. Our recent study suggested that the observed relationship between estrogens and SUA could be at least in part explained by the effect of these hormones on body fat distribution [10]. Estrogens promote the accumulation of adiposity in gluteo-femoral rather than in abdominal/visceral depots [20,21]. The dramatic decrease in estrogen levels, and the consequent increase of androgen (i.e., testosterone) to estradiol ratio occurring after menopause, leads to a fat distribution shift during menopause transition making postmenopausal women more prone to android (central) obesity.

In the present study, we focused on the potential effect of adiposity distribution on the association between SUA and bone health, in a cohort of premenopausal and postmenopausal women. To that end, we evaluated body fat amount and distribution using the gold-standard technique DXA and by BMI and WC, the two anthropometric parameters most commonly considered in epidemiological/clinical studies. On the other hand, bone health was determined by the measurement of BMD and well-accepted serum markers of bone turnover such as bone-specific alkaline phosphatase (BAP), C-terminal telopeptide of type I collagen (CTX-1), receptor activator of nuclear factor-κb ligand (RANKL) and osteoprotegerin (OPG).

## 2. Results

### 2.1. General Characteristics, Adiposity Indexes, BMD at Selected Skeletal Sites, Bone Markers and Uric Acid in the Study Sample

As shown in Table 1, osteoporosis was diagnosed in 63 postmenopausal women (111 showed osteopenia), while none of the younger premenopausal subjects were found to be affected by this disease (55 showed osteopenia). All the indexes of overall/central/peripheral adiposity as assessed by anthropometry or DXA were higher in the postmenopausal group compared to the premenopausal group. Conversely, BMD values of the lumbar spine, total hip, femoral neck and trochanter were all lower in the older postmenopausal women compared to those in reproductive age. Finally, SUA did not significantly differ between the two groups (Table 1).

### 2.2. Correlations of SUA with BMD and Bone Markers

Potential associations between SUA level and BMD at all sites were first evaluated in the whole study sample by bivariate correlation analysis revealing a weak positive correlation with SUA and BMD at the total hip (*r* = 0.120, *p* < 0.05). To check if menopausal status could influence the association between the purine catabolite and BMD, we performed the correlation test separately among premenopausal (*n* = 124) and postmenopausal (*n* = 234) women. The analysis revealed that SUA was significantly and positively associated with BMD at the total hip (*r* = 0.165, *p* < 0.05) and at the trochanter (*r* = 0.151, *p* < 0.05) among estrogen-deficient women but not among young women in the reproductive age (Supplementary Table S1). Possible SUA correlates were analyzed also among bone markers revealing only a significant inverse association with RANKL levels (*r* = −0.181, *p* < 0.05), which remained almost unaltered after adjustment for age, hormonal treatment, years since menopause, smoking status and indexes of adiposity.

### 2.3. Multivariable Association between SUA and Total Hip BMD

As the next step of our analysis, we evaluated whether the bivariate correlations of SUA with BMD at the trochanter and with BMD at the total hip were independent of potential confounding factors. After initial adjustment for age, hormonal treatment, years since menopause and smoking status, only the association between SUA and total hip BMD retained its significance (*β* = 0.220, *p* = 0.001). Since SUA showed highly significant correlations with both BMD and adiposity measures (Table 2), we evaluated the possible influence of BMI, WC and DXA indexes of fat mass (FM) on SUA vs. total hip BMD association.

As disclosed in Table 3, each inclusion of adiposity indexes in the multivariable model markedly affected the strength of the association between SUA and total hip BMD, maintaining a marginal significance only in the model with legs FM and percentage of trunk, legs or total FM (*p* < 0.05, for all models). On the contrary, after adjustment for BMI, WC, trunk FM or total FM the targeted relationship was no longer significant.

**Figure 1:**
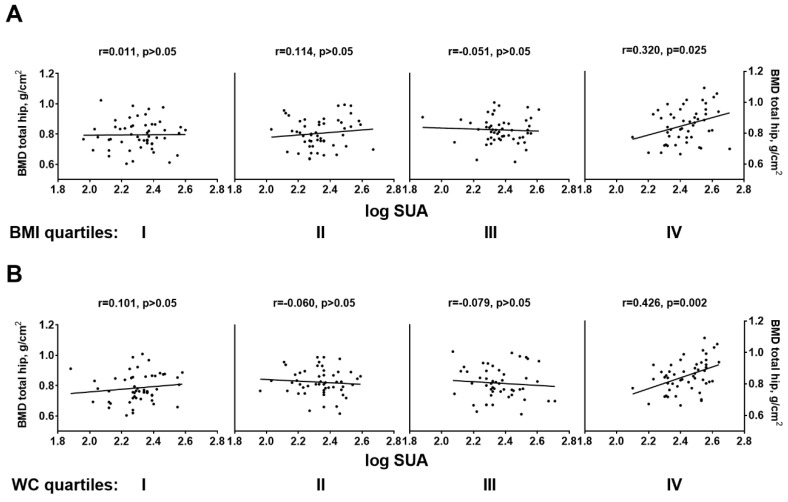
Pearson bivariate correlations between BMD at total Hip and logarithmic transformed—SUA across quartiles of BMI (**A**) and waist circumference (**B**) *r* = Pearson’s correlation coefficient. BMI quartiles: I, <22.4 kg/m^2^; II, 22.4–24.1 kg/m^2^; III, 24.2–26.7 kg/m^2^; IV, >26.7 kg/m^2^. Waist circumference (WC) quartiles: I, <78 cm; II, 78–84 cm; III, 84.1–91 cm; IV, >91 cm.

The strong influence of overall and central adiposity we observed was even more evident when the association between SUA and total hip BMD was explored within quartiles of BMI and WC. As displayed in Figure 1 and Supplemental Table S2, SUA and total hip BMD were significantly correlated only within the highest quartile of both anthropometric measures (panel (A), BMI quartile IV: *r* = 0.347, *p* = 0.02; panel B, WC quartile IV: *r* = 0.444, *p* = 0.001).

Different from what was observed in the whole sample and in postmenopausal subsamples, in the highest quartile of WC (but not in that of BMI) the targeted association retained its significance also after controlling for WC itself (*β* = 0.339, *p* = 0.033) or BMI (*β* = 0.333, *p* = 0.023) (data not shown). On the contrary, the association was no longer significant adding trunk FM or total FM as a covariate.

## 3. Discussion

In the present study we examined the possible association between SUA and surrogate markers of bone health in a sample of premenopausal and postmenopausal women to shed light on the controversial data reported in the literature on this topic. We found that, in postmenopausal women higher SUA was significantly related to an increased total hip BMD independent from age, hormonal treatment, smoking and years since menopause.

There is wide agreement on the indication that SUA could act as a systemic antioxidant, stimulating several studies to address its protective role against OS-related diseases. However, no conclusive evidence has been achieved so far demonstrating that the in vitro ability to scavenge ROS and protect cell membranes from lipid oxidation has a solid impact in vivo on humans [12,16]. Moreover, SUA is a “victim” of a paradox, since there is also evidence suggesting that it can act as a pro-oxidant, mostly in a free radicals-rich environment [12,16,22]. In some authors’ view this dark side of SUA is in line with the results of numerous studies linking high SUA levels with increased risk of atherosclerosis and related disorders [23,24]. In contrast with these apparent deleterious effects, some experimental and epidemiological studies have nonetheless suggested that SUA may protect against bone loss. In particular, there are some large population-based studies, with either cross-sectional or longitudinal design, supporting the view of SUA as a beneficial factor for bone health. The most supportive evidence comes from investigations on postmenopausal women and on men. A cross-sectional study, including 7502 healthy postmenopausal women found that serum SUA levels were positively associated with BMD at all sites [13]. In this line, similar results were those reported by Ishii et al. (sample: *n* = 615) [14] and more recently by Yan et al. (sample: *n* = 4256) [15]. In particular, in the latter study SUA was found to be positively correlated with BMD also in males (sample: *n* = 943) and associations with similar strengths and direction were reported in two other studies dealing with male subjects [25,26].

On the other hand, Zhang et al. found no independent association between SUA and BMD at any skeletal sites in the general population (6759 participants of National Health and Nutrition Examination Survey, NHANES; 2005–2010) [16]. Consistently, Lee et al. and Sritara et al. reported no correlation between SUA and BMD in postmenopausal and premenopausal women, respectively [25,27].

The authors of epidemiological studies usually explain the discrepancy between their findings and those of others by differences in study design, targeted sample population, sample structure and size as well as in covariates. We believe that selection of covariates included in the multivariable models assessing the relationship between SUA and BMD is a critical step that might account for the high variability of the published results on the topic. In our study, we did not consider potential important confounders such as glomerular filtration rate (GFR), which greatly influence SUA circulatory levels, or key regulatory factors (such as parathyroid hormone, vitamin D etc.) of bone metabolism. Nonetheless, to the best of our knowledge this is the first report assessing potential effects of DXA-derived indexes of total/regional adiposity and among the few to assess the influence of WC on the SUA vs. BMD relationship.

DXA is technically the superior of anthropometric-based measurements since it is able to discriminate fat from fat-free soft tissue [28,29]. Nevertheless, this radiological technique is also more expensive and less suitable for epidemiological studies; thereby BMI and WC remain the most common indexes of adiposity used in this context. Despite the aforementioned evidence on the preferential link between central fat and SUA, only BMI or body weight have been considered in the multivariate analysis in, at least, the largest studies supporting the independent association of the purine end-product and with BMD [13,14,27,30,31]. Conversely, in the present study, the association between SUA and BMD at the total hip was lost upon adjustment for BMI. In our view, a possible explanation for this discrepancy could lie in differences in WC range between ours and other population samples. To address this hypothesis, we examined the association between the two variables of interest across WC quartiles. Noteworthy, we found that the association was significant and independent from BMI (and WC) but not from DXA-derived trunk and total FM, only in the highest quartile.

Osteoporosis develops less often in men than in women and it has been considered for a long time as a “woman’s disease”, such as other complex diseases that have been stereotypically considered “men’s diseases”, creating a strong gender gap in patients’ health care [32,33,34,35]. However, osteoporosis in men has now been recognized as a key health issue. Females and males differ in terms of adipose tissues distribution with males inclined to accumulate more visceral/abdominal fat leading to the classic android body shape, whereas females tend to accumulate fat in the gluteo-femoral depot (gynoid pattern of fat distribution). The estrogen decline occurring during menopausal transition leads to weight gain and to a shift in fat distribution, making women body shape more similar to that of men. It is well known that this change is accompanied by a dramatic increase in cardiometabolic risk factors, including SUA. In contrast, body adiposity appears to improve bone health, and this effect might be mediated by the endocrine properties of adipocytes, physiological adaptation of the skeleton to the increased load, and by the effects of selected adipokines (e.g., adiponectin) [36,37]. It is tempting to speculate that SUA, through its antioxidant activity, might be one of the mediators of this beneficial effect of central adiposity on bone. A protective effect that becomes detectable only in postmenopausal women showing a more pronounced android pattern of fat distribution, which was absent in premenopausal women of our sample. In agreement with our results, Sritara et al. found a positive correlation between SUA and BMD in males and in a group of prevalently young females [25].

We acknowledge that the consistency of our data might be affected by the lack of consideration of important confounders such as glomerular filtration rate (GFR), physical activity and common female disorders (e.g., polycystic ovary syndrome) which greatly influences SUA circulatory levels and/or bone metabolism. However, to the best of our knowledge we were the first to evaluate the possible confounding effect of DXA-derived indexes of total/regional adiposity and, more surprisingly, among the few to check the influence of WC on SUA vs. BMD relationship.

In conclusion, the present study reports the association between SUA and total hip BMD in postmenopausal women, with central fat influencing this interplay. The results suggest that SUA may be beneficial for bone health in postmenopausal women showing a more android fat distribution, ascribing to SUA a targeted role during menopause transition that could also be considered and investigated in men in further targeted longitudinal studies.

## 4. Materials and Methods

### 4.1. Subjects

Data examined in this retrospective investigation were obtained from subjects randomly enrolled among women undergoing bone densitometry evaluation at the Centre of Menopause and Osteoporosis of the University of Ferrara (Ferrara, Italy), and among female employers of the University of Ferrara as described elsewhere [3,28]. The original purpose of the research protocol was to investigate possible relationships between menopause, related disturbances and diseases (including osteoporosis) and body FM distribution, by including in the analysis circulating markers of oxidative stress and inflammation. The Ethics Committee of the University of Ferrara approved the two research studies (REC numbers: CE 110291 and CE 130292) from which the data presented here were obtained. The research protocol was carried out in accordance with the Declaration of Helsinki (World Medical Association, http://www.wma.net). Written informed consent was obtained from each subject during the first visit at baseline before possible inclusion in the study. Eligible participants were Caucasian and apparently healthy women aged between 25 and 65 years, experiencing premenopausal or postmenopausal status. Menopausal status was defined according to the ReSTAGE’s modification of the Stages of Reproductive Aging Workshop (STRAW) staging criteria [38]. In line with these criteria, women reporting a regular menstrual cycle were regarded as in reproductive age (premenopausal status); women reporting amenorrhea longer than 12 months were regarded as in postmenopausal status. The most important exclusion criteria were pregnancy, breastfeeding, excessive alcohol intake (more than 20 g/day), concomitant chronic kidney or liver diseases, cardiovascular diseases (CVD), cancer, use of medications interfering with uric acid metabolism. Only three premenopausal women were diagnosed with osteoporosis (see the diagnostic criteria in the next paragraph) and 20 with diabetes that were excluded from the analysis for statistical reasons. Finally, 358 women were found to be eligible and were enrolled in the study.

### 4.2. Measures of Anthropometric and DXA Indexes of Adiposity

Body weight, standing height and WC were assessed by trained personnel and used to calculate values of BMI and WC according to standard protocols [28]. Body composition was measured by DXA using a QDR 4500 W apparatus (Hologic Inc., Marlborough, MA, USA). The software used for the instrument provided estimates of absolute (grams) and percentage of lean tissue mass, FM and bone mineral mass for the total body and for standard body regions. Using specific anatomical landmarks, regions for head, arms, trunk and legs were distinguished as reported elsewhere [39].

### 4.3. Bone Densitometry Assessment

Areal bone density was assessed at the lumbar spine, hip, and total body by a Discovery DXA scanner (Hologic Inc.). Osteoporosis was diagnosed when the BMD T-score (number of standard deviations below the average for a young adult at peak bone density) was lower than 2.5 standard deviations from BMD peak at either the femoral neck or lumbar spine, according to World Health Organization (WHO) guidelines [40]. In accordance with these criteria, women with a T-score at either the skeletal area between −2.5 and −1.0 were classified as osteopenic and those with a value higher than −1.0 as normal.

### 4.4. Biochemical Assays

Fasting blood samples were collected from each participant and serum was obtained after 30 min of incubation at room temperature and subsequent centrifugation (3000× *g* for 10 min). Serum was then aliquoted and stored at −80 °C until analysis. Commercially available Enzyme-Linked Immunosorbent Assays (ELISAs) kits were performed according to the manufacturer’s instructions and using a Tecan Infinite M200 microplate reader (Tecan group Ltd., Männedorf, Switzerland). The markers measured in serum were the following: total (free plus bound) soluble RANKL (by Human sRANKL (total) ELISA from BioVendor Research and Diagnostic Products, Modrice, Czech Republic); OPG (OPG ELISA, from Boster Biological Technology Co., Ltd., Wuhan, China); BAP (by OCTEIA Ostase BAP immunoenzymometric assay, from Immunodiagnostic Systems Ltd., Boldon, UK); CTX-1 (by serum Cross-Laps ELISA kit from Immunodiagnostic Systems Ltd. Boldon, UK).

Serum uric acid was determined by the direct enzymatic method [41] in which uric acid was oxidized by uricase coupled with peroxidase and the results were measured spectrophotometrically (intra-assay CV: 2.5%, inter-assay CV: 5.3%).

### 4.5. Statistical Analysis

Values were expressed as either mean ± SD or median and interquartile range when they were normally or non-normally distributed, respectively (data distribution were checked by Kolmogorov–Smirnov and Shapiro–Wilkinson tests). In case of non-normal variables, base-10 log transformation was used to approach the Gauss distribution. Categorical variables were expressed as a number and percentage within the group. The main characteristics of premenopausal and postmenopausal women were compared by performing one-way analysis of variance (ANOVA) (using Sidak as post-hoc test for pairwise comparison) or Kruskall–Wallis (with Mann–Whitney U test) for normally or non-normally distributed variables respectively. A chi-square test was used to compare differences in categorical variables. Simple correlation analyses were performed using Pearson’s and Spearman’s coefficients of correlation tests for normally and non-normally distributed variables, respectively. These analyses were used to evaluate potential associations of SUA with BMD at various skeletal sites or bone serum markers, and that of SUA and BMD with DXA-derived and anthropometric measures of adiposity in premenopausal and postmenopausal women. Multiple linear regression analysis was carried out to estimate whether the association between SUA and BMD at the trochanter and BMD at the total hip were independent of potential confounding factors such as age, smoking, hormonal treatment, and in once a time and one by one, an index of adiposity. The covariates referring to regional and overall adiposity were not included simultaneously in the regression model because of the collinearity issue (values of variance inflation factor above 2.5 were considered indicative of the presence of this statistical issue). Two-tailed probability values <0.05 were considered statistically significant.

## Figures and Tables

**Table 1:** Main characteristics of premenopausal and postmenopausal women included in the study sample.

Demographic Parameters	Premenopausal Women (*n* = 124)	Postmenopausal Women (*n* = 234)
Age, years	35 ± 10	55 ± 4 ^b^
Years since menopause, years	-	4.0 ± 0.1
Hormone treatment, % *	29	3 ^a^
Smokers, %	27	11 ^a^
Osteoporosis, %	0	27 ^a^
Anthropometric parameters and DXA indexes of body fat distribution		
BMI, kg/m^2^	23 ± 4	25 ± 3 ^b^
Waist circumference, cm	77 ± 10	85 ± 9 ^b^
Trunk FM, kg	7.8 ± 4	10.2 ± 3.8 ^a^
Legs FM, kg	7.7 ± 2.4	8.6 ± 2.3 ^a^
Total FM, kg	18.4 ± 6.9	22.3 ± 6.1 ^a^
Markers of bone health		
Lumbar spine BMD, g/cm^2^	0.99 ± 0.11	0.89 ± 0.09 ^a^
Total hip, BMD, g/cm^2^	0.89 ± 0.10	0.82 ± 0.09 ^a^
Femoral neck BMD, g/cm^2^	0.79 ± 0.11	0.70 ± 0.10 ^a^
Trochanter BMD, g/cm^2^	0.67 ± 0.10	0.62 ± 0.09 ^a^
CTX-1, ng/mL	-	0.64 ± 0.40
BAP, µg/L	-	20.9 ± 6.8
RANKL, pmol/L	-	334.1 ± 251.9
OPG, pmol/L	-	226.8 ± 147.3
RANKL/OPG	-	2.7 ± 4.3
Uric acid, µmol/L	225 ± 76	239 ± 76

Continuous data are expressed as mean ± standard deviations. Categorical data are expressed as *n*—(% in the group). * Current hormonal treatment includes contraceptives or hormone replacement therapy. Abbreviations: BMI, body mass index; FM, fat mass; BMD, bone mineral density; CTX-1, C-terminal telopeptides of Type I; BAP, bone-specific alkaline phosphatase; RANKL, receptor activator of nuclear factor-*κ*b ligand; OPG, osteoprotegerin. Difference between groups: ^a^ *p* < 0.05; ^b^ *p* < 0.001.

**Table 2:** Correlation (expressed as Pearson’s correlation coefficient) of serum uric acid (SUA) and bone mineral density (BMD) at the lumbar spine, total hip, femoral neck and trochanter with dual x-ray absorptiometry (DXA)-derived and anthropometric measures of adiposity in premenopausal and postmenopausal women.

Variables	Premenopausal Women (*n* = 124)					Postmenopausal Women (*n* = 234)				
	SUA	Lumbar Spine BMD	Total Hip BMD	Femoral Neck BMD	Trochanter BMD	SUA	Lumbar Spine BMD	Total Hip BMD	Femoral Neck BMD	Trochanter BMD
BMI	−0.013 *	0.227 ^a^	0.252 ^a^	0.190	0.261 ^a^	0.345 ^b^	0.264 ^b^	0.298 ^b^	0.240 ^b^	0.254 ^b^
WC	0.036	0.088	0.190	0.108	0.206	0.369 ^b^	0.286 ^b^	0.271 ^b^	0.270 ^b^	0.251 ^b^
Trunk FM	0.048	0.145	0.147	0.076	0.162	0.390 ^b^	0.285 ^b^	0.321 ^b^	0.291 ^b^	0.269 ^b^
Arms FM	0.007	0.128	0.134	0.078	0.157	0.292 ^b^	0.278 ^b^	0.327 ^b^	0.283 ^b^	0.266 ^b^
Legs FM	0.010	0.098	0.061	0.044	0.035	0.251 ^b^	0.162 ^a^	0.282 ^b^	0.293 ^b^	0.192 ^a^
Total FM	0.092	−0.017	−0.057	−0.113	−0.062	0.275 ^b^	0.241 ^b^	0.260 ^b^	0.219 ^b^	0.119
Total FM %	0.076	0.002	−0.072	−0.104	−0.062	0.365 ^b^	0.159 ^a^	0.233 ^b^	0.223 ^b^	0.140
Trunk FM %	0.105	0.043	−0.011	−0.058	−0.024	0.330 ^b^	0.211 ^b^	0.236 ^b^	0.202 ^b^	0.207 ^a^
Legs FM %	0.023	−0.104	−0.188	−0.188	−0.197	−0.202 ^a^	−0.055	0.084	0.123	−0.040

* Pearson’s correlation coefficient, r; Abbreviations: BMI, body mass index; FM, fat mass; BMD, bone mineral density; WC, waist circumference. ^a^ *p* < 0.05, ^b^ *p* < 0.001.

**Table 3:** Effect of surrogate markers of total and regional fat mass on the association between serum uric acid and BMD at the total hip in postmenopausal women.

Multiple Regression Models	Covariate Added to Model 1	β (R^2^ of Regression Model, %)	% Contribution of Total Hip BMD in Uric Acid Variance
1	-	0.220 ^a^ (17.6)	5.2
2	BMI	0.119 (24.0)	1.6 ↓↓ *
3	Waist circumference	0.119 (23.0)	1.5 ↓↓
4	Trunk FM	0.100 (22.1)	1.0 ↓↓
5	Legs FM	0.173 ^a^ (22.1)	3.4 ↓
6	Total FM	0.101 (26.1)	1.2 ↓↓

Model 1: outcome variable = total hip BMD; explanatory variable = uric acid, covariates: age, smoking, hormone treatment and years since menopause. Model 2–9: as for model 1 plus one of the surrogate markers of fat mass as an additional covariate. β = standardized regression coefficient for uric acid vs. total hip BMD. ^a^ *p* < 0.05. * Symbols indicating the change in the relative contribution of Total hip BMD in SUA variance compared to model 1: ↓ decrease by 25–50%; ↓↓ decrease by more than 50%. Abbreviations: BMI, body mass index; FM, fat mass; BMD, bone mineral density.

## Supplementary Materials

The following are available online at https://www.mdpi.com/1422-0067/20/24/6321/s1, Supplementary Table S1: Simple correlation coefficients (r) for the relationship between serum uric acid and BMD at different skeletal sites or serum markers of bone health among premenopausal and postmenopausal women. Supplementary Table S2: Unadjusted and adjusted association (expressed as standardized regression coefficient) between uric acid and total hip BMD across quartiles of BMI, waist circumference, trunk FM, and total FM.
