# Supplementary material for: Changes in Adipose Tissue Distribution and Association between Uric Acid and Bone Health during Menopause Transition
Source: Int J Mol Sci. 2019 Dec 14;20(24):6321. doi: 10.3390/ijms20246321 (PMC6941025; doi:10.3390/ijms20246321)
Supplement: Supplementary file 1 [file ijms-20-06321-s001.pdf]

International Journal of *Molecular Sciences*

MDPI

## Supplementary material

**Supplemental Table 1**. Simple correlation coefficients (r) for the relationship between serum uric acid and BMD at different skeletal sites or serum makers of bone health among premenopausal and postmenopausal women.

| **Bone parameters** | **Premenopausal women** (n = 124) | **Postmenopausal women** (n = 234) |
|---|---|---|
| Log Lumbar spine BMD | -0.082 | 0.056 |
| Total hip, BMD | -0.010 | 0.165 [a] |
| Femoral neck BMD | -0.043 | 0.126 |
| Log Trochanter BMD | -0.015 | 0.151 [a] |
| Log CTX-1 | - | -0.081 |
| Log BAP | - | 0.010 |
| Log RANKL | - | -0.181 [a] |
| Log OPG | - | -0.115 |
| Log RANKL/OPG | - | -0.059 |

a $p<0.05$. BMD at trochanter and lumbar spine, and all serum markers of bone health were log-transformed to approach normal distribution. Abbreviations: BMI, body mass index; FM, fat mass; BMD, bone mineral density; CTX-1, C-terminal telopeptides of Type I; BAP, bone-specific alkaline phosphatase; RANKL, receptor activator of nuclear factor-κb ligand; OPG, osteoprotegerin (OPG)

**Supplemental Table 2.** Unadjusted and adjusted association (expressed as standardized regression coefficient) between uric acid and total hip BMD across quartiles of BMI, waist circumference, trunk FM, and total FM

| **-Independent variables** | **Quartile I** | **Quartile II** | **Quartile III** | **Quartile IV** |
|---|---|---|---|---|
| -BMI (kg/m²) | <22.4 | 22.4-24.1 | 24.2-26.7 | >26.7 |
| β | -0.001 | 0.227 | 0.051 | 0.320 [a] |
| $\beta_{adjusted}$ | -0.002 | 0.228 | 0.004 | 0.347 [a] |
| -Waist circumference (cm) | <78 | 78-84 | 84.1-91 | >91 |
| β | 0.139 | 0.080 | -0.079 | 0.426 [a] |
| $\beta_{adjusted}$ | 0.137 | 0.101 | -0.006 | 0.444 [b] |
| -Trunk FM (Kg) | <7.4 | 7.4-9.8 | 9.9-12.6 | >12.6 |
| β | -0.040 | -0.070 | 0.201 | 0.286 [a] |
| $\beta_{adjusted}$ | -0.090 | 0.070 | -0.143 | 0.404 [a] |
| -Total FM (Kg) | <18.0 | 18.0-22.2 | 22.3-26.1 | >26.1 |
| β | 0.007 | 0.043 | -0.047 | 0.426 [a] |
| $\beta_{adjusted}$ | 0.048 | 0.011 | 0.032 | 0.445 [b] |

β= standardized regression coefficient for uric acid vs. total hip BMD. β adjusted= standardized regression coefficient for uric acid vs. total hip BMD after adjustment for age, smoking, hormones treatment and years since menopause. Abbreviations: BMI, body mass index; FM, fat mass; BMD, bone mineral density.

© 2019 by the authors. Submitted for possible open access publication under the terms and conditions of the Creative Commons Attribution (CC BY) license (http://creativecommons.org/licenses/by/4.0/).
